# Comparison of the Modified CTOD Measurement Method with the Double Clip Gauge Method in a Compact Tension Specimen

**DOI:** 10.3390/ma18020310

**Published:** 2025-01-11

**Authors:** Jeong Yeol Park, Myung Hyun Kim, Chang Wook Ji

**Affiliations:** 1 Smart Forming Process Group, Korea Institute of Industrial Technology, Ulsan 44776, Republic of Korea; cwji@kitech.re.kr; 2 Department of Naval Architecture and Ocean Engineering, Pusan National University, Busan 46241, Republic of Korea; kimm@pusan.ac.kr

**Keywords:** fracture toughness, CTOD, double clip gauge, rotational factor, compact tension specimen

## Abstract

For allowable defect analyses, the fracture toughness of materials needs to be accurately predicted. In this regard, a lower fluctuation of fracture toughness can lead to reduction in safety and economic risks. Crack tip opening displacement (CTOD), which is the representative parameter for fracture toughness, can be measured by various methods, such as the $\delta_{5}$, the J-conversion method, the single clip gauge method, and the double clip gauge method. When calculating CTOD from test results, the principle of similar triangles, which adopts the plastic hinge model, is influenced by the rotation factor, $r_{p}$. Therefore, in order to reduce the fluctuation of CTOD, the exact value of $r_{p}$ must be defined. This study investigates various methods to predict fracture toughness in metallic materials, and assess the pros and cons of each method. Moreover, the equation of $r_{p}$ is modified by using a double clip gauge in compact tension (CT) to reduce the fluctuation of CTOD. The $r_{p}$ value is derived from 0.55 to 0.68, using the double clip gauge method. Finite element analysis is used to derive the $r_{p}$ values, which range from 0.50 to 0.66, in order to verify the validity of the derived $r_{p}$ values. This ensures the validity of the $r_{p}$ value derived from the experiment. In addition, the fluctuation of CTOD, based on the modified equation of $r_{p}$, is lower than that using the single clip gauge method, according to BS 7448.

## 1. Introduction

A structural integrity assessment (SIA) should be performed before manufacturing or operating a welded structure. To assess its structure integrity, a fracture toughness test must be carried out. For allowable defect analyses in a SIA, in particular, fracture toughness needs to be accurately predicted. If the fracture toughness of a material has high fluctuation in each test, we should choose the minimum fracture toughness value to obtain safety results of the SIA. The conservative results of the SIA come from a minimum fracture toughness value. In this regard, lower fluctuation can lead to a reduction in the economic and safety risks.

Fracture toughness is usually used as a generic term for measurement of a material’s resistance to the extension of a crack [1]. Fracture toughness may be expressed in various parameters, such as K factor, J-integral, and crack tip opening displacement (CTOD), etc. K factor was developed by Irwin in 1957 to explain the intensity of elastic crack tip fields [2]. To describe the intensity of elastic–plastic crack tip fields, the J-integral was proposed by Rice in 1968 [3]. CTOD was developed by Wells in 1963, in order to function as an engineering fracture parameter [4]. Generally, the CTOD, which is the representative parameter for fracture toughness, has been used for elastic–plastic materials [5]. Until recently, various methods of evaluating CTOD have been developed [6,7,8,9,10]. The representative CTOD evaluation methods include J-conversion method and single clip gauge.

In addition, ASTM E1820 provides the equation which calculates CTOD from a *J* integral [11]:

(1) $$\delta =\frac{J}{m\sigma_{flow}}$$

where the m-factor is as following:

$$m=A_{0}+A_{1}\left(\frac{\sigma_{YS}}{\sigma_{TS}}\right)+A_{2}{\left(\frac{\sigma_{YS}}{\sigma_{TS}}\right)}^{2}-A_{3}{\left(\frac{\sigma_{YS}}{\sigma_{TS}}\right)}^{3}\;A_{0}=3.18-0.22\left(\frac{a_{0}}{W}\right),\;A_{1}=4.32-2.23\left(\frac{a_{0}}{W}\right),\;A_{2}=4.44-2.29\left(\frac{a_{0}}{W}\right),\;\mathrm{and}\;A_{3}=2.05-1.06\left(\frac{a_{0}}{W}\right)$$

where $\sigma_{YS}$ is the yield strength, and $\sigma_{TS}$ is the tensile strength. The observable advantage in this method is that two parameter of fracture toughness can be obtained using only one test. However, the method of ASTM E1820 underestimates the CTOD, unlike the direct measurement method of $r_{p}$ proposed in this study, because the CTOD is derived using conversion, based on the J integral [12].

In the single clip gauge method, a plastic hinge model was adopted for estimating the critical $\delta_{c}$ from CMOD measurements.

**Figure 1:**
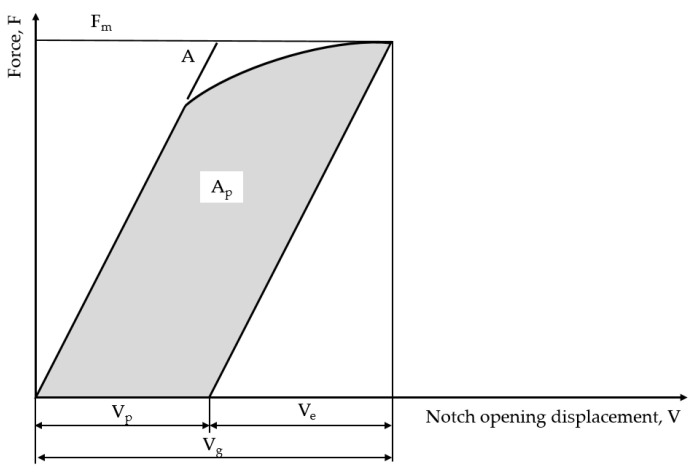
The definition of $V_{p}$.

The calculations of the CTOD of any point on the force–displacement curve (Figure 1) are made using the following expression [13]:

(2) $$\delta ={\left[\frac{F}{BW^{0.5}}f^{\prime }\left(\frac{a_{0}}{W}\right)\right]}^{2}\frac{\left(1-\nu^{2}\right)}{2\sigma_{YS}E}+\frac{0.46\left(W-a_{0}\right)V_{p}}{0.46W+0.54a_{0}+\left(C-W\right)+z}\;for\;CT$$

This method has the advantage of being easy to obtain the CTOD value by a simple test. In terms of the disadvantage, the CTOD values from this method have fluctuation in each test. The clip gauge method, commonly used to calculate fracture toughness, determines the CTOD using the principle of similar triangles in the visibility of a crack tip. When calculating CTOD from test results, the principle of similar triangles, which adopts the plastic hinge model, is influenced by the rotation factor, $r_{p}$. Therefore, in order to reduce the fluctuation of CTOD, the exact value of $r_{p}$ must be defined. In this study, CTOD was calculated using a variable $r_{p}$, rather than a fixed $r_{p}$, to reduce the fluctuation that occurs with repeated fracture testing.

This study suggests that the equation of $r_{p}$ should be modified by using a double clip gauge in compact tension (CT) to reduce the fluctuation of CTOD. Among the different experimental assessment methods for CTOD, we focused on the clip gauge method due to its simplicity. In order to validate the modified $r_{p}$ using the double clip gauge method, the experimental results in this study were compared to those of finite element analysis (FEA) and other referenced data.

## 2. Materials and Methods

### 2.1. Experimental Set up

The material considered for a CTOD test was Al 5083-O(UACJ Corporation, Tokyo, Japan). The chemical composition of Al 5083-O is presented in Table 1. Al 5083-O must contain levels of Cu and Zn below 0.1%, because they are known to harm its erosion resistance. Moreover, Al 5083-O must contain Si below 0.3% due to its detrimental effect on fracture toughness [14]. Mg also affects the mechanical properties of Al 5083-O. If Al 5083-O contains Mg over 5%, the quality of its mechanical properties is known to deteriorate [14]. The tensile test for Al 5083-O was conducted based on ASTM E8 [15]. The crosshead speed in this study was 1 mm/min. The mechanical properties of Al 5083-O are summarized in Table 2. As shown in Table 2, tensile tests are performed a total of 10 times, and the test results are summarized in a range.

**Figure 2:**
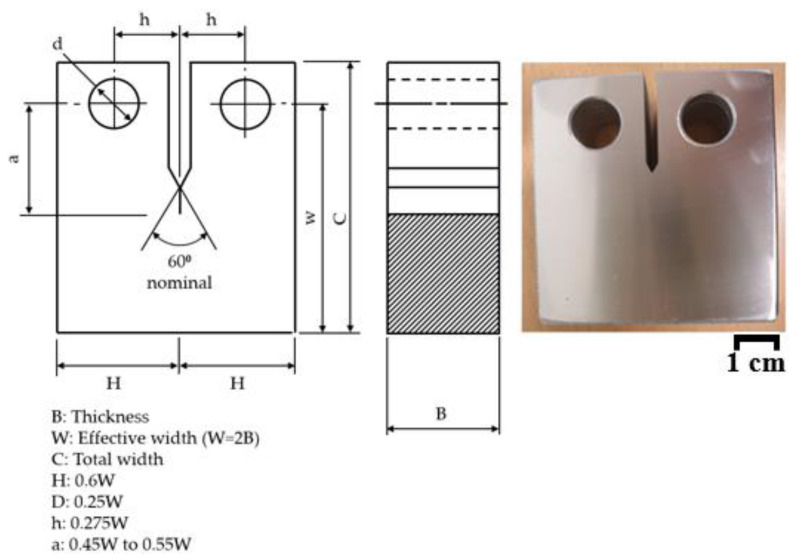
CTOD test of a CT specimen using the double clip gauge method.

In the case of the single clip gauge method, a CTOD test was conducted according to BS 7448 [13]. In order to calculate the modified $r_{p}$, CTOD tests were carried out based on the double clip gauge method. As shown in Figure 2, we performed the CTOD test using a CT specimen. In this study, *W* is 50 mm and *B* is 25 mm. The length of the fatigue pre-crack is 2.5 mm. The test conditions are summarized in Table 3.

**Figure 3:**
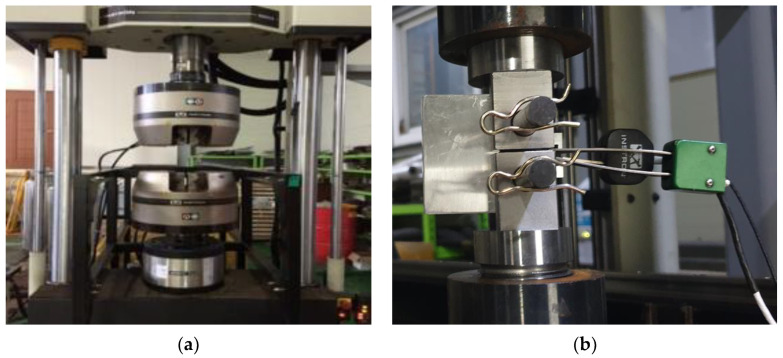
Test machine: (**a**) Servo hydraulic test machine (Instron Model 8803); (**b**) COD gauges (Instron and Epsilon).

The test machine used for the tensile, fatigue, and fracture tests was a servo hydraulic testing machine (Instron, High Wycombe, UK) with a maximum load capacity of ±50 ton and a COD gauge (Instron, High Wycombe, UK). The test machine is presented in Figure 3. Before the CTOD test, according to the double clip gauge method, we set the test specimen and COD gauges (Instron and Epsilon), as shown in Figure 3.

### 2.2. Double Clip Gauge Method

**Figure 4:**
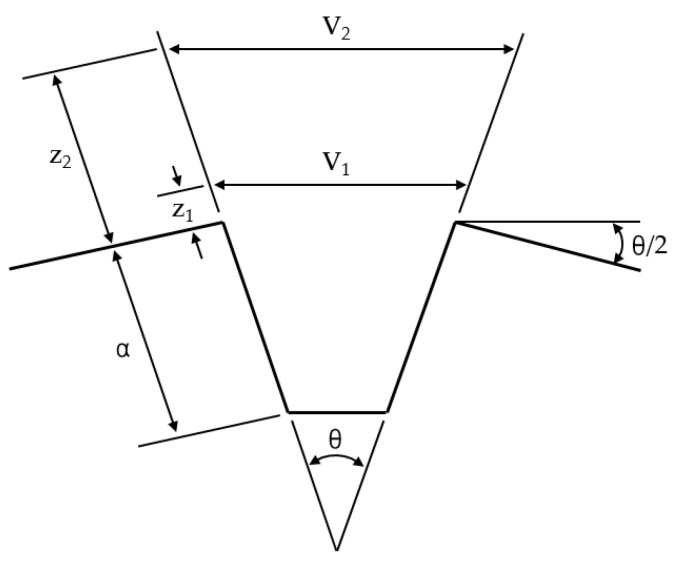
Principle of similar triangles in the visibility of a crack tip.

**Figure 5:**
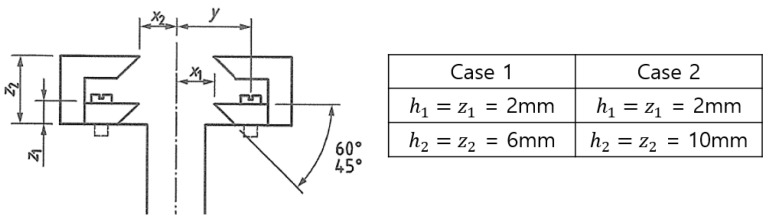
Dimension of the knife edge.

As shown in Figure 4, a double clip gauge, which is similar in principle to a single clip gauge, determines the CTOD using the principle of similar triangles in the visibility of a crack tip [5]. When calculating CTOD from test results, the principle of similar triangles, which adopts the plastic hinge model, is influenced by the rotation factor, $r_{p}$. In addition, the double clip gauge method was used for the knife edge and two COD gauges. Double clip gauges were mounted to the specimen surface with two heights, as shown in Figure 5.

(3) $$\delta ={\left[\frac{F}{BW^{0.5}}f^{\prime }\left(\frac{a_{0}}{W}\right)\right]}^{2}\frac{\left(1-\nu^{2}\right)}{2\sigma_{YS}E}+V_{1}-\frac{a_{0}+h_{1}}{h_{2}-h_{1}}\left(V_{2}-V_{1}\right)$$

where $V_{1}$ and $V_{2}$ are the plastic parts of the clip gauge displacements. As mentioned above, in case of the double clip gauge, CTOD values are directly inferred, using the linear extrapolation method from the double gauge measured displacements, following the rigid rotation assumption. Accordingly, CTOD can be estimated from a similar triangles construction from the double clip gauges [5]. In terms of calculating the $r_{p}$, the test is conducted by different conditions of the knife edge to determine the sensitivity, according to the height of the knife edge. When applying the double clip gauge method to the SENT specimen, Park et al. used a gauge location of 2 mm ($z_{1}$) and 8 mm ($z_{2}$), and compared it with a single clip gauge to obtain reasonable results [16]. In this study, as shown in Figure 5, $z_{1}$ is manufactured with 2 mm and $z_{2}$ with 6 mm in case 1, and $z_{1}$ is conducted with 2 mm and $z_{2}$ with 10 mm in case 2. In addition, the sensitivity is assessed by applying each case to a CT specimen.

### 2.3. Finite Element Analysis

**Figure 6:**
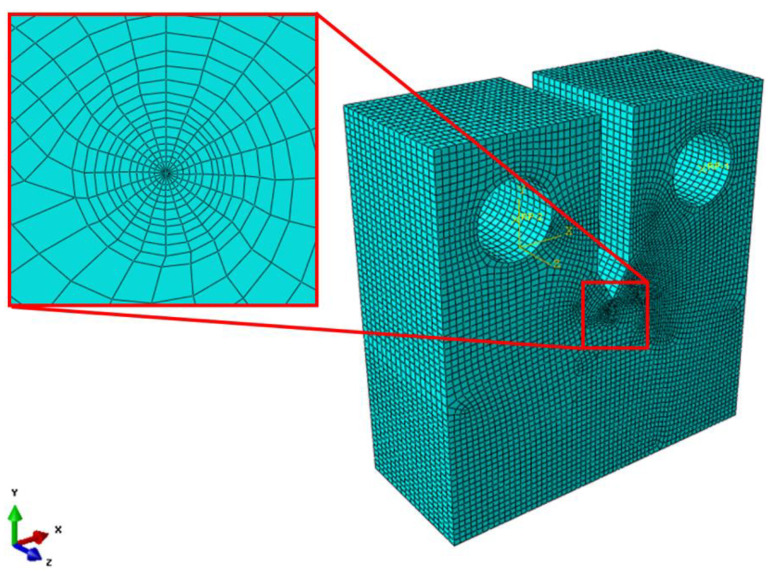
The finite element analysis model of CT specimen.

To validate the CTOD using the modified $r_{p}$, the CTOD was obtained by FEA (using Abaqus). As shown in Figure 6, we conducted modeling and meshing of a CT specimen. The ratio of *a/W* was set at 0.5 in the specimen model. For the mesh type, an 8-node (linear type) reduced-integration mesh (C3D8R) for 3D stress analysis was applied, the crack tip was modeled in a wedge format, and C3D6, which was fixed to 6 nodes due to the geometric characteristics of the element, was used. In addition, the number of meshes was 80,410, and the Ramberg–Osgood model from Equation (4) was applied to the material properties for plasticity analysis. The Ramberg–Osgood model is commonly used for the representation of the stress–strain curve of materials [17]. In addition, the idealization of material properties using the Ramberg–Osgood model is required to derive fracture toughness using finite element analysis [18]. Therefore, in this study, the material properties were idealized by deriving the material constants of the Ramberg–Osgood model, based on the actual tensile test results of Al 5083-O. The analysis was performed by the displacement controlled loading on the rigid hole surface. Moreover, after finite element analysis, CTOD was estimated by applying the 45° intercept method to the center of the thickness of the test specimen [19].

(4) $$\frac{\varepsilon }{\varepsilon_{0}}=\frac{\sigma }{\sigma_{0}}+\alpha {\left(\frac{\sigma }{\sigma_{0}}\right)}^{n}$$

where $\sigma_{0}$ is reference stress, which is yield stress, $\varepsilon_{0}$ is reference strain, α and n are material constants.

## 3. Results and Discussion

**Figure 7:**
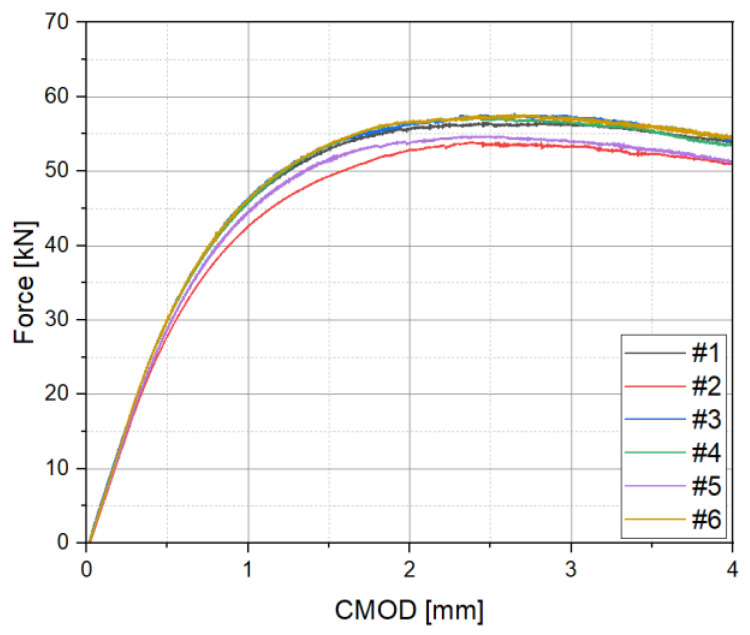
PV curves, based on single clip gauge method.

A CTOD test was conducted by applying the single clip gauge method, based on BS 7448. To verify its reproducibility, the test was repeated a total of six times, and the CTOD was calculated based on $r_{p}$, which was fixed 0.46, and the PV diagram in Figure 7. As a result, as summarized in Table 4, the CTOD value was found to be 0.77 mm from 0.61 mm, and the deviation was confirmed to be 0.16 mm. It is assumed that the cause of the deviation of CTOD was the lack of sensitivity to plastic deformation, due to the fixed $r_{p}$ value, in the single clip gauge method.

### 3.1. Double Clip Gauge Method

#### 3.1.1. Modified $r_{p}$

Conventional CTOD measurement methods can lead to an increase in the fluctuation of CTOD values. In order to decrease fluctuation, double clip gauge displacements were used for the calculation of $r_{p}$. In the case of the existing double clip gauge method, the plastic part of CTOD was calculated through the principle of similar triangles, based on the P-$V_{1}$-$V_{2}$ diagram derived from the experiment. Taking this into consideration, the double clip gauge method and the principle of similar triangles were adopted to suggest a modified equation that calculates $r_{p}$ based on the PV diagram derived from the actual CTOD test results. Therefore, the $r_{p}$ calculation equations correspond to the CT specimen, which is Equations (5)–(8), and is presented in the following equation.

(5) $$\delta_{pl}=\frac{r_{p}\left(W-a_{0}\right)V_{p1}}{r_{p}W+\left(1-r_{p}\right)a_{0}+\left(C-W\right)+h_{1}}=V_{p1}-\frac{a_{0}+h_{1}}{h_{2}-h_{1}}\left(V_{p2}-V_{p1}\right)$$

(6) $$\left(r_{p}W-V_{p1}r_{p}W+V_{p1}a_{0}-V_{p1}C+V_{p1}w-V_{p1}h_{1}\right)\left(-h_{2}+h_{1}\right)\;=\left(r_{p}W-a_{0}-a_{0}r_{p}+\left(C-W\right)+h_{1}\right)\left(V_{p2}a_{0}+V_{p1}h_{1}+a_{0}V_{p1}+h_{1}V_{p1}\right)$$

(7) $$r_{p}\left(\left(V_{p1}\left(W\left(h_{2}-3h_{1}-a_{0}\right)-a_{0}\left(a_{0}+2h_{1}\right)\right)\right)-a_{0}V_{p2}\left(W-a_{0}\right)-W\left(h_{2}+h_{1}\right)\right)\;=\left(V_{p1}\left(h_{2}-3h_{1}-a_{0}\right)-a_{0}V_{p2}\right)\left(W+a_{0}-C-h_{1}\right)$$

(8) $$r_{p}=\frac{\left(V_{p1}\left(h_{2}-3h_{1}-a_{0}\right)-a_{0}V_{p2}\right)\left(W+a_{0}-C-h_{1}\right)}{\left(\left(V_{p1}\left(W\left(h_{2}-3h_{1}-a_{0}\right)-a_{0}\left(a_{0}+2h_{1}\right)\right)\right)-a_{0}V_{p2}\left(W-a_{0}\right)-W\left(h_{2}+h_{1}\right)\right)}$$

where $h_{1}$ and $h_{2}$ are the heights of the first and second knife edges, respectively, and $V_{p1}$ and $V_{p2}$ are the plastic displacement recorded at the first and second knife edges, respectively. $a_{0}$ is the initial crack length, *C* is the total width of the CT specimen, and *W* is the width up to the load line displacement (LLD) of the CT specimen.

#### 3.1.2. Results of CTOD Based on Modified $r_{p}$

**Figure 8:**
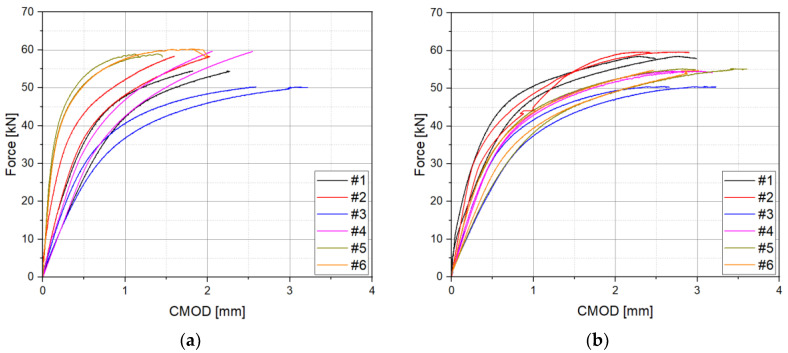
P-$V_{1}$-$V_{2}$ curves: (**a**) case 1 ($h_{1}$ = 2 mm, $h_{2}$ = 6 mm); (**b**) case 2 ($h_{1}$ = 2 mm, $h_{2}$ = 10 mm).

**Figure 9:**
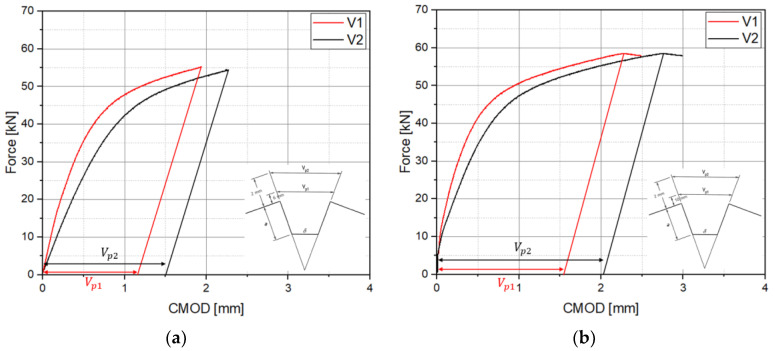
Calculation of $V_{p1}$ and $V_{p2}$ based on P-$V_{1}$-$V_{2}$ curves: (**a**) case 1 ($h_{1}$ = 2 mm, $h_{2}$ = 6 mm); (**b**) case 2 ($h_{1}$ = 2 mm, $h_{2}$ = 10 mm).

Fracture toughness tests were conducted six times for each case by applying the double clip gauge method, and the P-$V_{1}$-$V_{2}$ diagram is shown in Figure 8. The maximum load (Fm) was approximately 55 kN to 60 kN, and no pop-in occurred. In addition, a representative P-$V_{1}$-$V_{2}$ diagram, based on the experimental results at case 1 and case 2, is shown in Figure 9. The modified CTOD evaluation method calculates the $V_{p1}$ and $V_{p2}$ based on the slope of the elastic part of $V_{1}$ and $V_{2}$, which is derived by applying the double clip gauge method.

As mentioned above, the $r_{p}$ value was derived using the calculated $V_{p1}$ and $V_{p2}$ and Equation (8), and is shown in Table 5. As shown in Table 5 and Figure 9, the difference between $V_{1}$ and $V_{2}$ is 0.31 mm for case 1 and 0.43 mm for case 2, based on the average value, so it increases by about 27% as $h_{2}$ increases. In addition, $r_{p}$ of case 1 is found to be from 0.63 to 0.68, and the range of $r_{p}$ for case 2 is 0.75 to 0.80. Therefore, it is confirmed that, as the difference between $h_{2}$ and $h_{1}$ increases, the calculated $r_{p}$ is larger. This means that, as the difference in location for the clip gauge increases, the difference in CMOD increases, and the $r_{p}$ value, which affects the plastic part, grows, the fracture toughness of the material is predicted to be higher. It is believed that the overestimation of the fracture toughness of a material can lead to dangerous results for the structure, by allowing a crack size that is larger than the actual crack size when predicting the allowable crack size within the SIA procedure.

**Figure 10:**
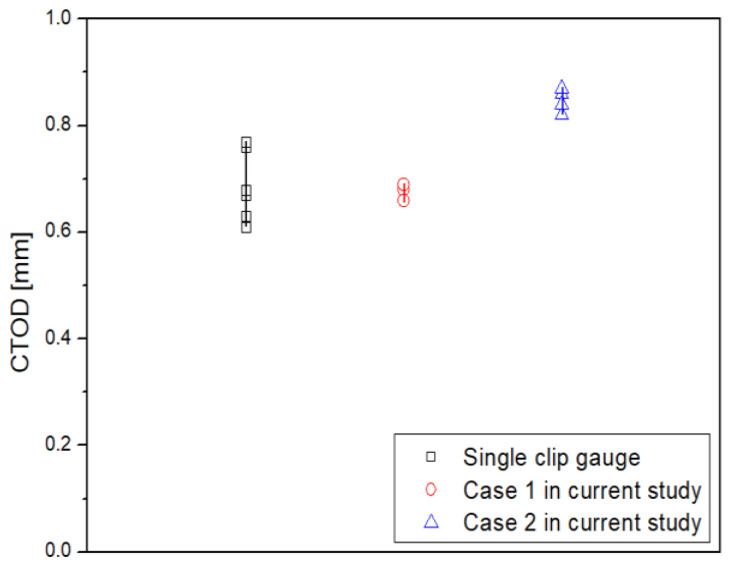
Comparison of CTOD between single and double clip gauge methods.

Based on the comparison of the derived $r_{p}$, the CTOD is calculated for case 1, which is an appropriate condition, and the results are compared with the CTOD derived using the existing method in Figure 10 [20]. The CTOD of case 1 is found to be from 0.66 mm to 0.69 mm, and the deviation occurred is approximately 0.03 mm. As a result, the CTOD deviation of the double clip gauge method decreased by about five times compared to the existing method. Therefore, the modified $r_{p}$ calculation equation that increased the plastic deformation sensitivity reduced the CTOD deviation.

### 3.2. Validation and Comparison of $r_{p}$ Using Finite Element Analysis and Other Studies

**Figure 11:**
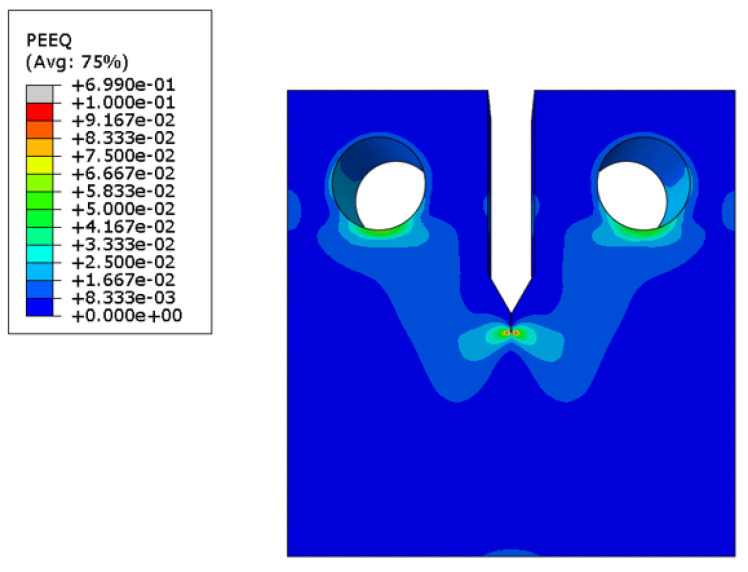
Contour maps of the equivalent plastic strains for a CT specimen.

To verify the effectiveness of $r_{p}$ derived using the double clip gauge method, finite element analysis results in quasi-static displacement control conditions, as are shown in Figure 11. As a result, the CTOD at the center of the test specimen thickness is about 0.69 mm, which is similar to the average value of the experimental results, and $r_{p}$ is calculated using Equation (9) [21]:

(9) $$r_{p}=\frac{x_{0}}{W-a_{0}}$$

where $x_{0}$ is the distance from the crack tip where the plastic strain in the y direction becomes 0.

**Figure 12:**
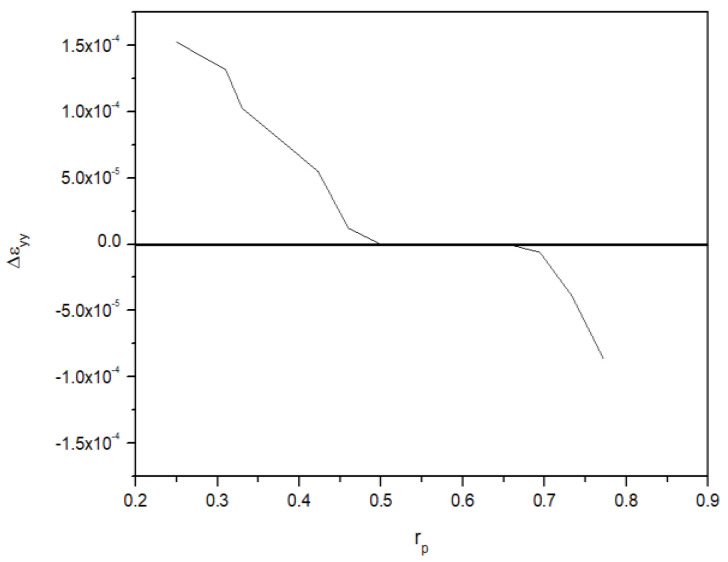
The relationship between the plastic deformation by yy direction and $r_{p}$ in a CT specimen.

In Figure 12, the $r_{p}$ derived using Equation (9) are summarized in relation to the plastic strain in the yy direction. When calculating CTOD using the plastic hinge model, $r_{p}$ must be considered. As the CMOD in the test specimen increases due to an applied force, the upper part, using the criteria of $r_{p}$, has a tensile strain and the lower part, using the criteria of $r_{p}$, has a compressive strain. The strain in the $r_{p}$ part converges to 0 [22,23]. Therefore, in particular, the deformation in the yy direction is defined as the part where $r_{p}$ converges to 0. In case of the finite element analysis results, it appears as a range rather than a specific numerical value. As summarized in Table 6, when the CTOD is 0.77 mm, the $r_{p}$ values, obtained from the finite element analysis results, are 0.50–0.68. The $r_{p}$ values derived from the finite element analysis were confirmed to be similar to the $r_{p}$ range derived under case 1 conditions. Therefore, the location of the double clip gauge needs to be considered in the experiment when applying case 1 conditions. In addition, the error that occurs when compared to the actual test results is confirmed to be within about 5%. Therefore, in case 1, the $r_{p}$ and CTOD derived using the modified $r_{p}$ method produce reasonable results.

**Figure 13:**
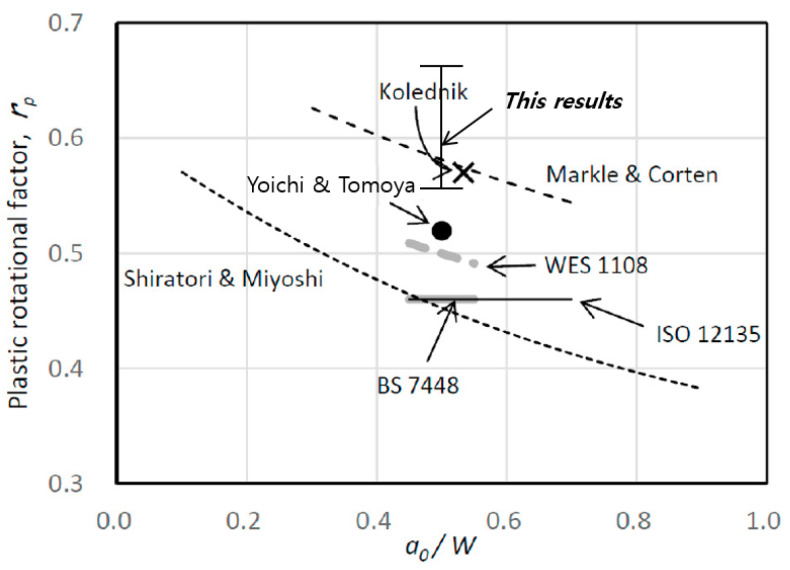
Comparison of $r_{p}$ between the current study and the existing data [13,21,24,25,26,27,28].

As shown in Figure 13, a comparison was conducted with previous research results related to $r_{p}$ [13,21,24,25,26,27,28]. The comparison revealed that, among the various $r_{p}$-related studies, the results of case 1 were similar to those of Yoichi and Tomoya [21], Kolednik [24], and Markle and Corten [25]. However, the $r_{p}$ value of case 2 showed higher numbers compared to other research results. In addition, it was confirmed that the studies by Shiratori and Miyoshi [26] and the standards, such as BS 7448 [13], WES 1108 [27], and ISO 12135 [28], predicted lower $r_{p}$ values, leading to a conservative estimation of CTOD. Therefore, as a method to minimize the safety margin of CTOD, it is appropriate to apply the double clip gauge method to calculate CTOD, with the $z_{2}$ value set to 6 mm.

## 4. Conclusions

The aim of this study is to analyze the modified $r_{p}$ using the double clip gauge method with a CT specimen, and compare this with the CTOD values of other CTOD assessment methods. Based on the results from this study, the following conclusions are drawn:

- To reduce the increase in fluctuation due to the increase in the number of tests, which is a shortcoming of the existing CTOD calculation method, a new calculation method for $r_{p}$, which affects the CTOD calculation, is proposed, as follows:
  $$r_{p}=\frac{\left(V_{p1}\left(h_{2}-3h_{1}-a_{0}\right)-a_{0}V_{p2}\right)\left(W+a_{0}-C-h_{1}\right)}{\left(\left(V_{p1}\left(W\left(h_{2}-3h_{1}-a_{0}\right)-a_{0}\left(a_{0}+2h_{1}\right)\right)\right)-a_{0}V_{p2}\left(W-a_{0}\right)-W\left(h_{2}+h_{1}\right)\right)}$$
  In addition, in order to derive a reasonable $r_{p}$ based on the improvement equation, the double clip gauge arrangement at the crack tip was optimized in the experiment as $h_{1}$ = 2 mm, $h_{2}$ = 6 mm.

- In the case of the calculation of CTOD using the proposed $r_{p}$, the value is 0.77–0.78, and the deviation is 0.01. Compared to the CTOD results (0.61–0.77) and deviation (0.16) derived from the existing single clip gauge, a reduction of approximately 90% is confirmed. Therefore, when the proposed $r_{p}$ equation is applied, it is verified that the fluctuation, according to the number of tests, is reduced.
- Comparisons are made between FEA results and the previous research to ensure the reliability of the proposed $r_{p}$ equation. The $r_{p}$ value, derived from the FEA, is found to be 0.50–0.68, and the $r_{p}$ value derived from the test results using the optimized double clip gauge arrangement conditions is confirmed to be 0.55–0.68. Therefore, the FEA results and the experimental results showed similar trends. The proposed $r_{p}$ is at least 23%, and up to 34% higher than the $r_{p}$ of the existing standards (ISO, BS and WES), but shows a similar trend when compared with the results of previous researchers.
- Finally, the fluctuation of CTOD values by the proposed $r_{p}$ using the double clip gauge method is lower than other methods. It is estimated that the modified $r_{p}$ can lead to a reduction in the economic and safety risks of the structural integrity assessment. However, the modified $r_{p}$ value is required to be used in more cases to reduce the prediction error, and further investigations should be carried out in the future.

## Figures and Tables

**Table 1:** Chemical composition of Al 5083-O.

Si	Fe	Cu	Mn	Mg	Cr	Zn	Ti	Cr-EQ
0.070	0.200	0.020	0.600	4.800	0.070	0.010	0.020	0.223

**Table 2:** Mechanical properties of Al 5083-O.

E [GPa]	$0.2\%\;\sigma_{YS}$ [MPa]	$\sigma_{TS}$ [MPa]
52–70	157–194	324–328

**Table 3:** Test conditions for CTOD.

*a/W*	Control Mode	Crosshead Speed [mm/min]
0.5	Displacement	1

**Table 4:** CTOD, based on single clip gauge method.

Test Number	CTOD [mm]
#1	0.61
#2	0.63
#3	0.67
#4	0.68
#5	0.76
#6	0.77

**Table 5:** $r_{p}$ based on the double clip gauge method.

Test Number	$r_{p}$	
	Case 1	Case 2
#1	0.63	0.80
#2	0.64	0.76
#3	0.55	0.75
#4	0.68	0.72
#5	0.67	0.80
#6	0.61	0.79

**Table 6:** Comparison of $r_{p}$ between experimental results and finite element analysis results.

Classification	$r_{p}$
Experimental results	0.55–0.68
Finite element analysis results	0.50–0.68

## Data Availability

The original contributions presented in the study are included in the article, further inquiries can be directed to the corresponding authors.
